# Isolation and Characterisation of a Recombinant Antibody Fragment That Binds NCAM1-Expressing Intervertebral Disc Cells

**DOI:** 10.1371/journal.pone.0083678

**Published:** 2013-12-13

**Authors:** Claire Cunningham, Akshay Srivastava, Estelle Collin, Sibylle Grad, Mauro Alini, Abhay Pandit, J. Gerard Wall

**Affiliations:** 1 Network of Excellence for Functional Biomaterials (NFB), National University of Ireland, Galway (NUI Galway), Galway, Ireland; 2 Microbiology, NUI Galway, Galway, Ireland; 3 AO Research Institute, Davos, Switzerland; Universidade do Porto, Portugal

## Abstract

Degeneration of the intervertebral discs (IVD) is a leading cause of neck and low back pain. Degeneration begins in the central nucleus pulposus region, leading to loss of IVD osmotic properties. Regeneration approaches include administration of matrix-mimicking scaffolds, cells and/or therapeutic factors. Cell-targeting strategies are likely to improve delivery due to the low cell numbers in the IVD. Single-chain antibody fragments (scFvs) that bind IVD cells were isolated for potential delivery of therapeutics to degenerated IVD. The most cell-distal domain of neural cell adhesion molecule 1 (NCAM1) was cloned and expressed in *Escherichia coli*. Phage display technology was used to isolate a human scFv against the recombinant domain by panning a scFv library on the immobilised protein. The isolated scFv bound cultured rat astrocytes, as well as bovine nucleus pulposus and annulus fibrosus cells in immunocytochemical studies. The scFv also labelled cells in bovine spinal cord and six-month and two-year old bovine IVD sections by immunohistochemistry. Antibody fragments can provide cell-binding moieties at improved cost, time, yield and functionalisation potential over whole antibodies. The described scFv has potential application in delivery of therapeutics to NCAM1-expressing cells in degenerated IVD.

## Introduction

Degeneration of the intervertebral discs (IVD) is a leading causative factor of neck and low back pain [1]. It has a lifetime incidence in excess of 70% [2] and is a main source of disability and lost workdays in industrialised nations. While it is particularly linked to aging, mild disc degeneration has been noted in as many as 20% of teenagers [3,4].

The intervertebral discs are located between the vertebral bodies in the spinal column and transmit loads arising from body weight and muscle activity. The IVD consist of a central gelatinous and highly hydrated nucleus pulposus (NP), bound peripherally by the annulus fibrosus (AF) and flanking cartilaginous end plates [5]. The NP is rich in extracellular matrix (ECM) proteins and is composed mainly of type II collagen and proteoglycans, predominantly aggrecan, whereas the fibrous AF consists of organised lamella composed mainly of type I collagen [6]. The ECM is produced and maintained by the IVD cells, which are markedly depleted in aged or degenerated tissue [7].

Disc degeneration begins in the NP with the progressive loss of proteoglycans, coupled with synthesis of type I collagen [1]. As a result, the NP becomes fibrotic and dehydrated, leading to loss of osmotic properties, reduction in disc height and, ultimately, painful and disabling pathophysiologies in the spinal column [5,8,9]. Current treatments are non-curative, typically alleviate pain only temporarily and many are highly invasive. Of these, non-surgical treatments include physiotherapy and the use of anti-inflammatory drugs. Surgical interventions, such as disc fusion or replacement, may affect spine biomechanics, can be suboptimal in delaying degeneration [10] and can even increase degeneration in neighbouring spinal segments [11].

Recent advances in IVD tissue engineering include the development of ECM-mimicking scaffolds [12-14] containing stem or disc-derived cells to replenish cell numbers [15,16] and/or protein- and DNA-based drugs to slow degeneration and/or stimulate resident cells [17]. Given the low density of resident IVD cells, cell-specific targeting of therapies is likely to enhance the efficacy of such treatment approaches.

Cell targeting typically exploits antibodies to target specific cells and to improve uptake of attached payloads [18,19]. Recombinant antibody fragments are antibody-derived molecules that retain the binding properties of their parent antibodies. They exhibit better tissue penetration and more rapid clearance than whole antibodies due to their reduced size and they can be easily engineered to add novel functions such as imaging or therapeutic moieties [20]. Single-chain antibody fragments (scFvs) consist of antibody heavy and light chain variable domains, joined by a peptide linker [20]. ScFvs specific for ligands of interest can be isolated from large, diverse scFv collections using a bacteriophage-based display technology that mimics the humoral response *in vitro* [21]. Importantly, this approach can provide entirely human proteins for *in vivo* applications. Recombinant fragments are also typically produced in bacterial hosts which are much more rapid and less expensive than animal or hybridoma technologies used in polyclonal or monoclonal antibody production.

Neural cell adhesion molecule (NCAM1; CD56) is an immunoglobulin superfamily member that acts as a receptor for intracellular signalling and plays an important role in embryogenesis and development [22]. It has been found to be upregulated in NP compared to AF cells and articular chondrocytes in canine [23] and human [24] tissues, indicating its potential usefulness as a target for delivery of therapeutics to IVD cells.

This study utilised a phage display and protein expression approach to successfully isolate a NCAM1-binding scFv *in vitro*. The isolated scFv bound NCAM1 expressing IVD cells and may be of interest for application in drug delivery systems specifically targeting IVD cells, envisaging disc regeneration.

## Materials and Methods

### Ethics Statement

Five-month-old and two-year-old calf bovine fresh tails were collected directly with permission after sacrifice of animals at Galway City Abattoir. Soft tissues surrounding IVDs (muscles and ligaments) were removed and cells and tissues were prepared as previously described [15]. All work was performed on explanted tissue and cells and, as biological material was harvested from practices undertaken for the purposes of recognised animal husbandry, did not require ethical approval under relevant Irish legislation (Statutory Instrument No. 543 of 2012). The Neu7 rat astrocyte cell line was a kind gift from Professor J. Fawcett, University of Cambridge and was cultured as previously outlined [25].

### Materials

Materials were purchased from Sigma-Aldrich (Ireland) unless otherwise stated. *Escherichia coli* strains TOP10 and W3110 were used to express the Ig1 domain of NCAM1 (NCAM1-Ig1), TG1 to propagate phage and HB2151 for scFv expression. The modified KM13 helper phage (MRC HGMP Resource Centre, Cambridge, UK) provided phage proteins for phagemid replication. The YamoI human scFv library was from Montarop Yamabhai, Suranaree University of Technology, Thailand [25]. The pIG6 vector for protein expression was from Andreas Plückthun, University of Zürich, Switzerland.

### Bioinformatic analysis and cloning

Human NCAM1 nucleotide sequence was retrieved from GenBank (BCO47244). The NCAM1-Ig1 domain structure (PDB ID 2NCM) [26] was analysed using DeepView Swiss-Pdb Viewer [27] (www.expasy.org/spdbv). The gene encoding NCAM1-Ig1 was amplified from human cDNA using ompA_NCAM1_F and NCAM1_R primers (Table S1), to add a C-terminal hexahistidine tag for protein detection and purification. The amplification product was combined by overlap PCR with an *E. coli ompA* leader sequence, for secretion to the *E. coli* periplasm, and FLAG motif for protein detection [28], which were amplified from pIG6 using ompA_F and ompA_NCAM1_R primers (Table S1). The combined product was cloned into the pIG6 vector and sequenced prior to expression. IgBLAST (www.ncbi.nlm.nih.gov/igblast/) was used to identify antibody variable genes homologous to isolated scFvs.

### Recombinant protein expression

NCAM1-Ig1 was expressed in *E. coli* W3110 cells containing pIG6/NCAM1-Ig1 using an auto-induction approach [29,30]. A 500-ml culture in ZYP-5052 medium was grown at 25°C with shaking for 24 h prior to harvesting of cells by centrifugation. After resuspension of cells in phosphate-buffered saline (PBS) and re-centrifugation, periplasmic proteins were extracted [31,32] and dialysed overnight against 5 l of immobilised metal affinity chromatography (IMAC) binding buffer (3.98 *M* NaCl, 80 mM NaH_2_PO_4_, 80 mM Na_2_HPO_4_.2H_2_O) at 4°C.

For expression of scFvs, phagemid DNA was extracted from overnight cultures and used to transform non-amber-suppressor *E. coli* HB2151 cells. A freshly transformed colony from TYE agar plates containing 1% glucose and 100 µg/ml ampicillin was used to inoculate 5 ml of LB containing glucose and ampicillin, followed by protein expression and extraction as described above.

### Protein purification

Protein purification was carried out by IMAC [33]. Tween-20 (2% v/v) and 10 mM imidazole were added to protein extracts before passing through a 1-ml HisTrap affinity column (GE Healthcare, UK) at 1 ml/min. The column was washed with 10 ml, 5 ml and 5 ml of binding buffer containing 20 mM, 50 mM and 80 mM imidazole, respectively, before elution using binding buffer containing 100 mM (scFvs) or 300 mM (NCAM1-Ig1) imidazole. Eluted fractions were dialysed as above and purified proteins were analysed by SDS-PAGE, immunoblotting [33] and enzyme immunoassay (ELISA; below).

### Isolation of NCAM1-Ig1-specific scFvs

Procedures for phage propagation and titration were as described previously [25,34]. Immunotubes were coated with 100 µg/ml NCAM1-Ig1 in PBS for 16 h at 4°C and blocked with 3% blocking agent (Rounds 1, 4: Marvel skimmed milk powder (Chivers, Ireland); Rounds 2, 5: Bovine serum albumin (BSA); Round 3: Ovalbumin) for 1 h at 37°C. After washing with PBS, 10^12^ phages in PBS containing 2% of the relevant blocking agent were added to each well. After incubation for 1 h at room temperature with rocking and 1 h without, wells were washed 20 times (8 PBS/0.1% Tween-20, 7 PBS/0.2% Tween-20, 5 PBS/0.5% Tween-20). Phages were eluted using 50 mM glycine-HCl (pH 2.0) to break the scFv-antigen interaction and 10 mg/ml trypsin to proteolyse phage particles from immobilised scFvs. Eluted phages were used to infect *E. coli* TG1 cells and rescued using KM13 helper phage for successive panning rounds [25]. Eluted phage populations were screened by ELISA for NCAM1-Ig1 binding after each panning round and phage particles isolated from individual bacterial clones were screened after round 4. Phagemid DNA was purified and scFv genes sequenced from clones with the highest NCAM1-binding signals. Candidate scFvs were expressed in soluble, non-phage-bound format and purified as described above.

### ELISA

Wells of a 96-well microtitre plate were coated overnight at 4°C with 100 µg/ml NCAM1-Ig1 in PBS. After three washes with PBS, wells were blocked using 2% skimmed milk powder for 2 h at room temperature. After three PBS washes, wells were incubated for 1 h at room temperature with scFvs (3.3 µg in 100 µl) that had been pre-incubated with 0-267 µg/ml NCAM1-Ig1. Wells were washed three times with PBS/0.1% Tween-20 and three times with PBS, followed by 1-h incubation at room temperature with an anti-myc horseradish peroxidase (HRP)-conjugated mouse IgG (Abcam, UK), diluted 1:1,000 in PBS, to detect the scFv *myc* tag [25]. After repeating the wash step, 3,3',5,5'-tetramethylbenzidine (TMB) substrate was added, reactions were stopped using 1 M H_2_SO_4_ and absorbances were read at 450 nm.

ELISA analysis of polyclonal (phage population-derived) and monoclonal (bacterial clone-derived) phage-displayed scFvs followed the above procedure with the exception that 50 μl of bacteriophage particles, diluted in blocking agent, replaced the soluble scFv and bacteriophages were detected using a HRP-conjugated mouse anti-M13 antibody (GE Healthcare), diluted 1:8,000 in PBS.

### Immunocytochemistry

Primary rat astrocytes were isolated [35] and seeded at 25,000 cells/cm^2^ on glass slides (Lab-Tek™ II Chamber Slide™ System, Nunc GmbH, Germany) coated with 0.01% (v/v) poly-L-lysine in Hank’s buffer salt solution. Cells were cultured for five days at 37°C to 70-80% confluence in DMEM/Ham’s nutrient mixture F12 (1:1) containing 1% penicillin-streptomycin and 10% fetal bovine serum.

NP and AF cells were extracted from two-year old bovine tails [15] and cultured in monolayer to an approximate confluence of 80%. After trypsinisation, cells were seeded at 25,000 cells/cm^2^ on glass slides (Lab-Tek™) and cultured for 48 h at 37°C in DMEM supplemented with 1% penicillin-streptomycin and 10% fetal bovine serum prior to fixing.

Rat astrocytes and bovine NP and AF cells were washed twice with PBS before fixing with 4% paraformaldehyde in PBS for 15 min and blocking with 1% goat serum in PBS for 30 min at room temperature. After incubation with 5 µg B5 scFv or control 2H12 scFv (10 µg/ml), scFvs were detected using a murine anti-polyhistidine monoclonal antibody (1:200 in PBS/0.1% goat serum), followed by a goat anti-mouse FITC-conjugated antibody (Molecular Probes, USA), diluted 1:500 in PBS/0.1% goat serum. A murine anti-human NCAM1 IgG1 (Abcam) was used as positive control at 1:250 in PBS/0.1% goat serum and detected using a goat anti-mouse FITC-labelled antibody (Molecular Probes), diluted 1:250 as above. Antibodies were incubated for 1 h at room temperature and three 5-min washes were carried out with PBS/0.1% Tween-20 after each incubation. Cell nuclei were stained using DAPI (1:10,000 in PBS) for 15 min at room temperature, followed by two 5-min washes with PBS. Imaging was performed using an Olympus IX81 inverted epifluorescent microscope.

### Immunohistochemistry

Bovine caudal spinal cord tissue was extracted from the spinal canal and harvested from two year-old bovine tail. Tissues were fixed with 4% paraformaldehyde in PBS. After three washes with PBS, tissues were infiltrated overnight with 20% sucrose, flash-frozen in liquid nitrogen-cooled isopentane and 5-µm frozen sections were cut on a Leica CM 1850 cryostat (Laboratory Instruments & Supplies Ltd., Ireland). Sections were collected on Superfrost^®^ Plus slides (Fisher Scientific Inc., Ireland) and stored at -20°C. Sections were washed three times with PBS prior to treating with 20 µg/ml (30 U/mg) proteinase K at 37°C for 5 min, followed by three further PBS washes. After blocking with PBS/2% BSA at room temperature for 1 h and three PBS washes, tissues were incubated with 5 µg B5 scFv or 5 µg of a commercial murine anti-NCAM monoclonal antibody (Sigma; 10 µg/ml) in 0.2% BSA overnight at 4°C. After three washes with PBS/0.1% Tween-20, a mouse monoclonal anti-polyhistidine IgG, diluted 1:50 in 0.2% BSA, was added to wells containing scFvs for 1 h at room temperature. Three PBS/0.1% Tween-20 washes were followed by incubation with an anti-mouse FITC-conjugated antibody (Invitrogen Technologies), diluted 1:500 in 0.2% BSA/PBS, for 1 h at room temperature. After three washes with PBS/0.1% Tween-20, cell nuclei were counterstained using DAPI (1:1,000 in PBS; Invitrogen Technologies) for 10 min at room temperature, followed by two washes with PBS. Fluorescence was preserved using Prolong^®^ Gold Anti-fade reagent (Invitrogen Technologies).

For IVD immunohistochemistry, soft tissues surrounding IVDs were removed from six-month and two-year old animals and IVD tissues were harvested. After collection of 10-µm thick transverse cryosections in glass slides as described above, sections were dried for 15 min at room temperature, washed three times with PBS and digested with proteinase K. Slides were washed three times with PBS and blocked with 5% goat serum for 1 h at room temperature. B5 scFv (500 µl of 10 µg/ml), diluted in PBS/0.1% goat serum, was incubated with sections overnight at 4°C. After five washes in 0.05% Tween-20, slides were incubated for 1 h at room temperature in turn with a rabbit anti-polyhistidine IgG and an anti-rabbit DyLight 568-conjugated antibody. Slides were counter-stained with DAPI, diluted 1:5000 in methanol, for 10 min at room temperature and washed three times with PBS/0.05% Tween-20 before preservation of fluorescence. In parallel analyses, the B5 scFv was pre-incubated with purified NCAM1-Ig1 at 50 µg/ml prior to investigation with sections.

## Results

### Cloning of recombinant NCAM1-Ig1

**Figure 1:**
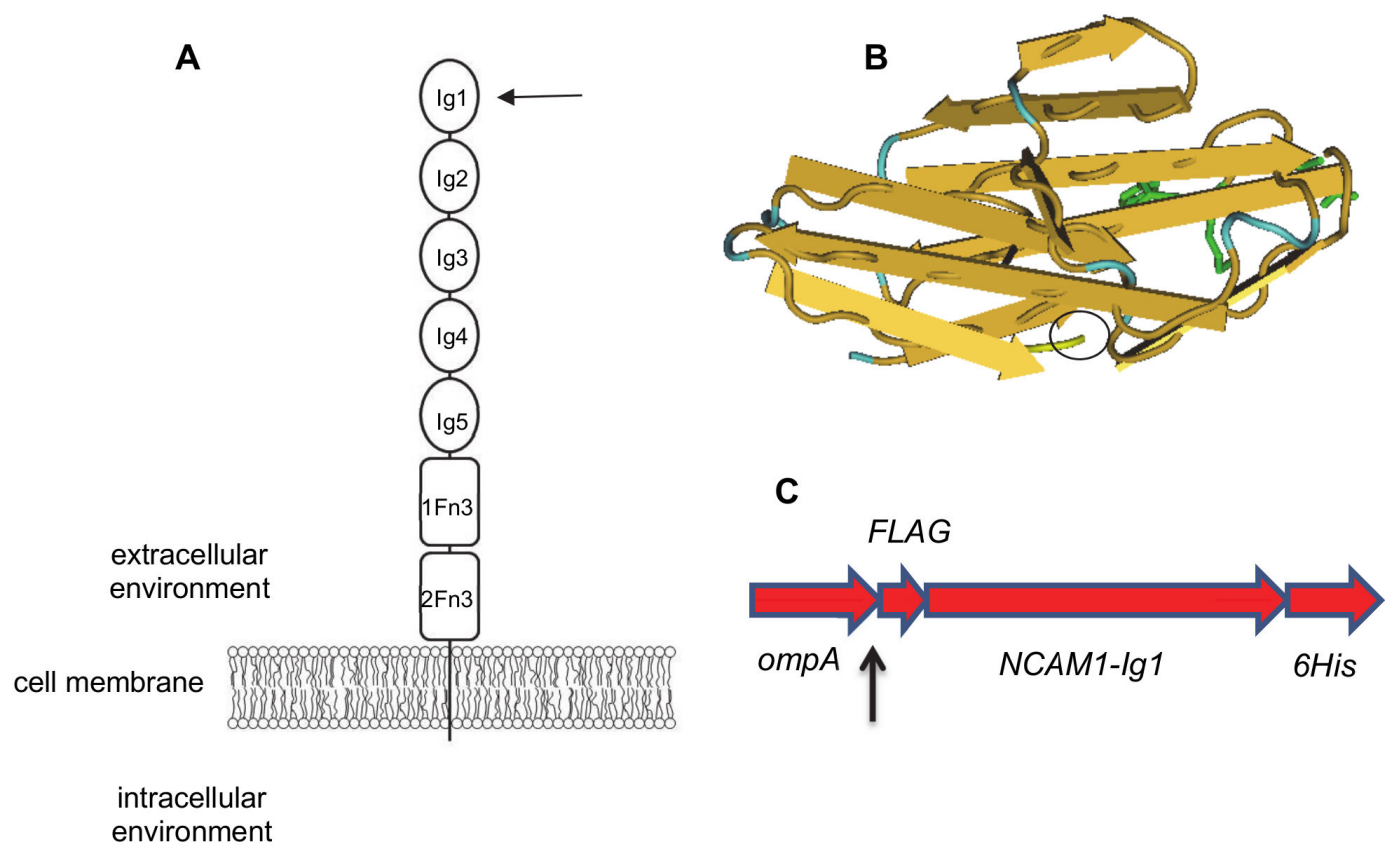
Schematic representation of the cloned NCAM1-Ig1 domain. A. Schematic of human NCAM1 structure. Immunoglobulin-like domains (Ig1-Ig5) are represented by ovals; fibronectin type III-like domains (1Fn3, 2Fn3) are shown as rounded rectangles. The most cell-distal, N-terminal Ig1 domain (arrowed), was cloned and expressed in *E. coli*. B. Crystal structure of NCAM1-Ig1 domain (PDB ID 2NCM) [26]. The C-terminal end, to which a hexahistidine peptide tag was attached for purification, is circled. Residues Glu179, Lys181 and Phe182 which are involved in the formation of the Ig1-Ig2 dimer interface are shown in green. C. Structure of the recombinant NCAM-Ig1 domain expressed in *E. coli*. *ompA* leader peptide for secretion to bacterial periplasm; this is cleaved (arrowed site) during translocation of the cytoplasmic membrane. FLAG: DYKD amino acid motif for protein detection. NCAM1-Ig1: gene encoding Ig1 domain of human NCAM1. 6His: hexahistidine tag for detection and purification of the recombinant NCAM1 domain.

NCAM1 contains five Ig-like modules and two fibronectin-like modules connected *via* a membrane-spanning domain to a signal-transducing cytoplasmic region (Figure 1). Based on GenBank sequence BCO47244, the sequence encoding the most cell-distal Ig-like domain, NCAM1-Ig1, was identified, amplified and cloned into the pIG6 expression vector. The resultant construct also contained an N-terminal *ompA* leader sequence for secretion of the polypeptide product to the *E. coli* periplasm, an adjacent FLAG tag for its detection [36], a C-terminal hexahistidine motif for IMAC-based purification and detection of the translated product, and flanking restriction enzyme sites. The confirmed sequence of the recombinant NCAM1-Ig1 domain is shown in Figure S1.

### Expression and purification

**Figure 2:**
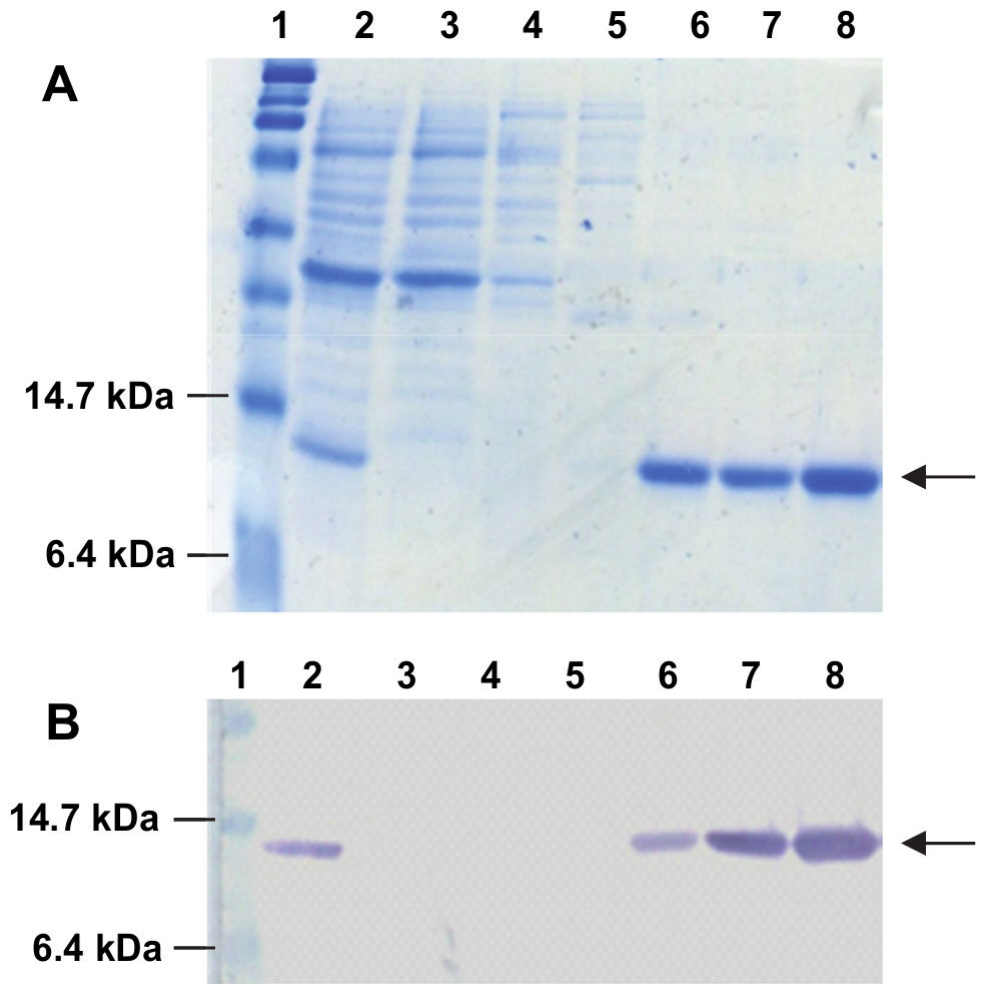
IMAC purification of recombinant NCAM1-Ig1 expressed in *E. coli*. (A) Coomassie stain and (B) western blot analysis of purification. Lane 1: molecular weight markers; lane 2: soluble periplasmic proteins from host bacterial cells; lane 3: IMAC column flow through; lanes 4-6: column washes with 20 mM, 50 mM and 80 mM imidazole; lanes 7, 8: proteins eluted using 100 mM imidazole. An anti-hexahistidine antibody was used to detect the target protein in (B). Arrows indicate proteins of the expected molecular weight (the predicted size of the NCAM1-Ig1 domain is 11.3 kDa).

Expression of recombinant NCAM1-Ig1 in *E. coli* TOP10 using IPTG induction generated low yields of predominantly insoluble protein (data not shown). Functional yields were increased by using *E. coli* W3110 and adopting an auto-induction protocol [29], whereby expression was induced following glucose depletion in the medium. This resulted in higher yields of NCAM1-Ig1 polypeptide and reduced proportions of insoluble protein. After isolation of soluble periplasmic proteins, 2-3 mg (determined using a Nanodrop 2000c; Thermo Scientific) of the 11.3-kDa hexahistidine-tagged NCAM1-Ig1 domain was purified to near-homogeneity by IMAC per litre of bacterial culture (Figure 2). Eluate fractions containing the highest protein concentrations were dialysed against PBS prior to use.

### Isolation of NCAM1-Ig1-specific scFvs

**Figure 3:**
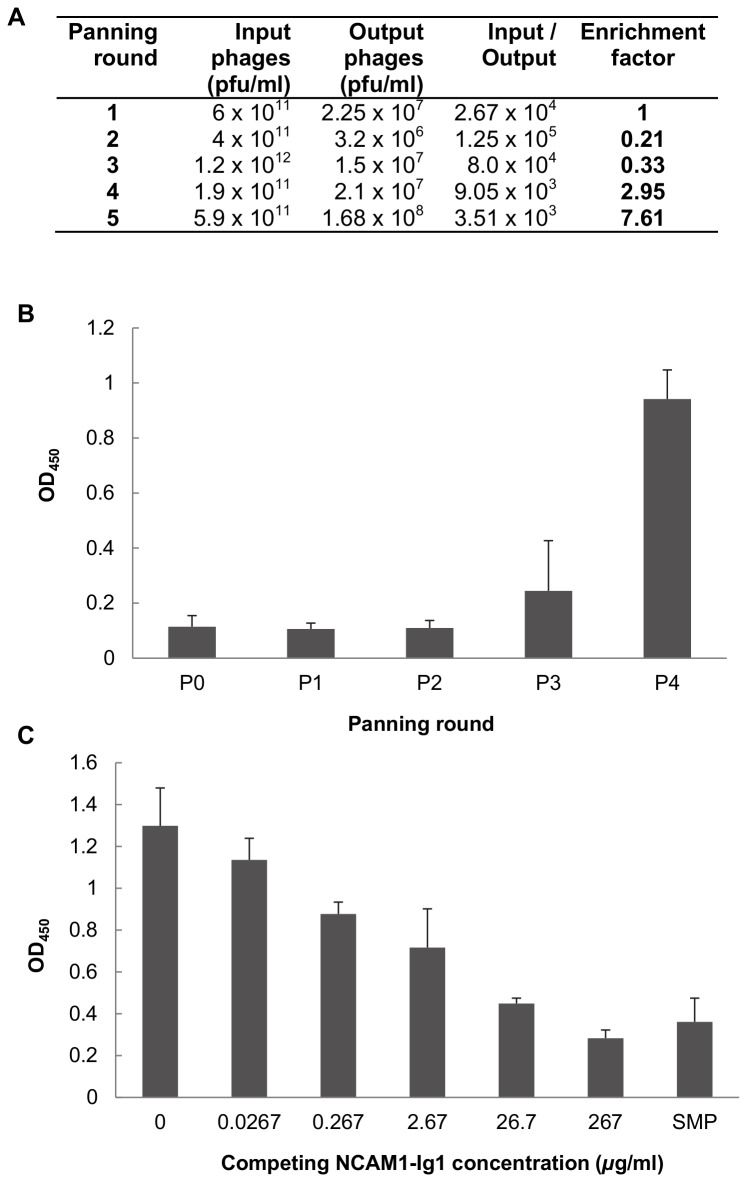
Isolation of NCAM1-binding scFvs by phage display. (A) Titres of input and output phage populations throughout library panning. Pfu = phage-forming units. (B) ELISA analysis of NCAM1-Ig1 binding activity of eluted polyclonal phage-scFv populations. P0: Unpanned library. P1-P4: Polyclonal phage preparations eluted after rounds 1 to 4 of library panning against immobilised NCAM1-Ig1. NCAM1-Ig1 was coated in ELISA wells at 100 µg/ml. Bars represent the mean of three replicate wells and error bars indicate the standard deviation of the mean. (C) Competitive ELISA analysis of NCAM1-Ig1 binding of B5 scFv. Binding of the purified scFv to immobilised NCAM1-Ig1 (100 µg/ml) was out-competed by pre-incubating the scFv with soluble NCAM1-Ig1 domain prior to ELISA analysis. SMP: skimmed milk powder control. Bars represent the mean of three replicate wells and error bars indicate the standard deviation of the mean.

Five rounds of panning of the YamoI human scFv phage library were carried out on immobilised NCAM1-Ig1.Washing stringency and antigen concentration were manipulated to enhance phage enrichment, which was identified after 4-5 panning rounds (Figure 3A). This correlated with a significant increase in NCAM1-Ig1 binding in eluted polyclonal phage populations after the fourth panning round (Figure 3B).

ELISA screening of phage-displayed scFvs from 160 randomly selected *E. coli* clones from panning round 4 identified eight NCAM1-binding molecules. DNA sequencing revealed two uninterrupted, full-length scFv gene sequences between six clones and intragenic stop codons in two scFvs. Based on these results, clones F4 and B5 from the former group were investigated by ELISA and B5 was selected for further analysis as it exhibited more efficient inhibition by free NCAM1-Ig1 domain in competitive ELISA (Figure 3C). The DNA sequence of the B5 scFv VH chain exhibited highest homology to human IGHV1-2 germline V gene using IgBLAST and nucleotide identities in excess of 90% to VH domains from human anti-Sm and anti-Epstein Barr virus LMP1 (latent membrane protein) antibodies using BLAST [37]. The B5 VL, meanwhile, exhibited highest homologies to germline IGLV6-57 V gene and IGLJ2 and IGLJ3 J genes, and sequence identities of 95% or greater to human Vλ genes from a number of anti-rabies virus antibodies.

### In vitro activity of B5 scFv

The B5 scFv was expressed in soluble, non-phage-bound form using the non-suppressor *E. coli* HB2151 strain to terminate translation at the amber stop codon between the scFv and the phage pIII protein [25]. After purification using IMAC, its binding of, and inhibition by, the purified NCAM1-Ig1 domain was confirmed by ELISA (Figure 3C).

**Figure 4:**
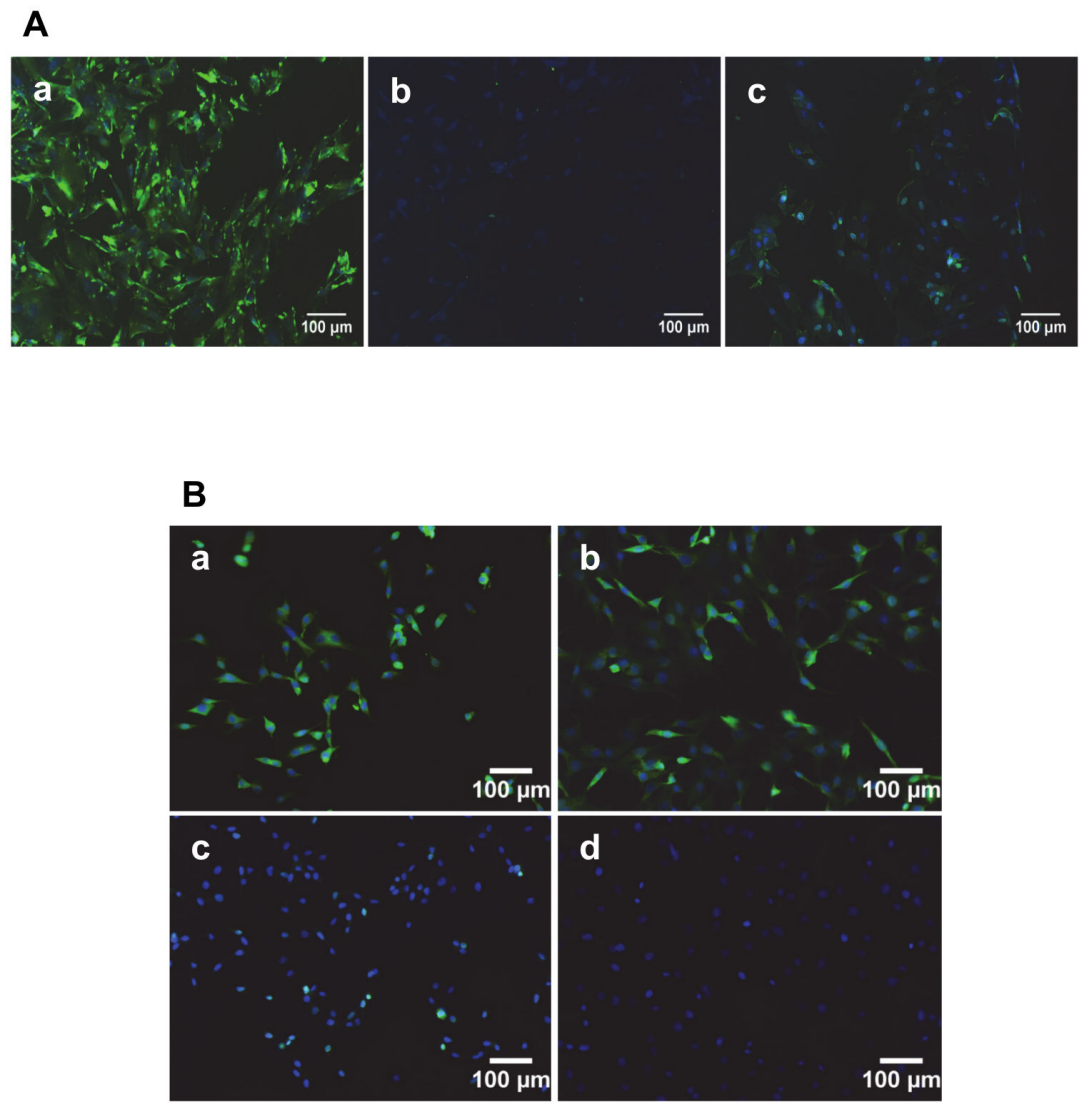
Immunocytochemical analysis of antibody binding to rat astrocytes and bovine IVD cells. A. Rat astrocytes were incubated with a. Anti-NCAM1 B5 scFv, b. Control 2H12 scFv, or c. Commercial anti-NCAM1 murine IgG, followed by detection using mouse monoclonal anti-histidine and goat anti-mouse-FITC reporter antibodies, respectively. B. Analysis of B5 scFv binding to bovine nucleus pulposus (a,c) and annulus fibrosus (b,d) cells. a,b: Cells labelled with B5 scFv, followed by appropriate reporter antibodies. c,d: As for a,b but no B5 scFv. Cell nuclei are stained blue using DAPI and scFvs attached to the cell surface are stained in green. Scale bar is 100 µm in all images.

The ability of B5 scFv to bind full-length NCAM1 protein on the cell surface was initially investigated using rat astrocytes, which have been reported to have high NCAM1 expression levels [22,23]. Cells were clearly labelled using the B5 scFv and not a control, neurotoxin-binding scFv [31,33], while a similar labelling pattern was observed using a commercial mouse anti-human NCAM1 IgG (Figure 4A).

**Figure 5:**
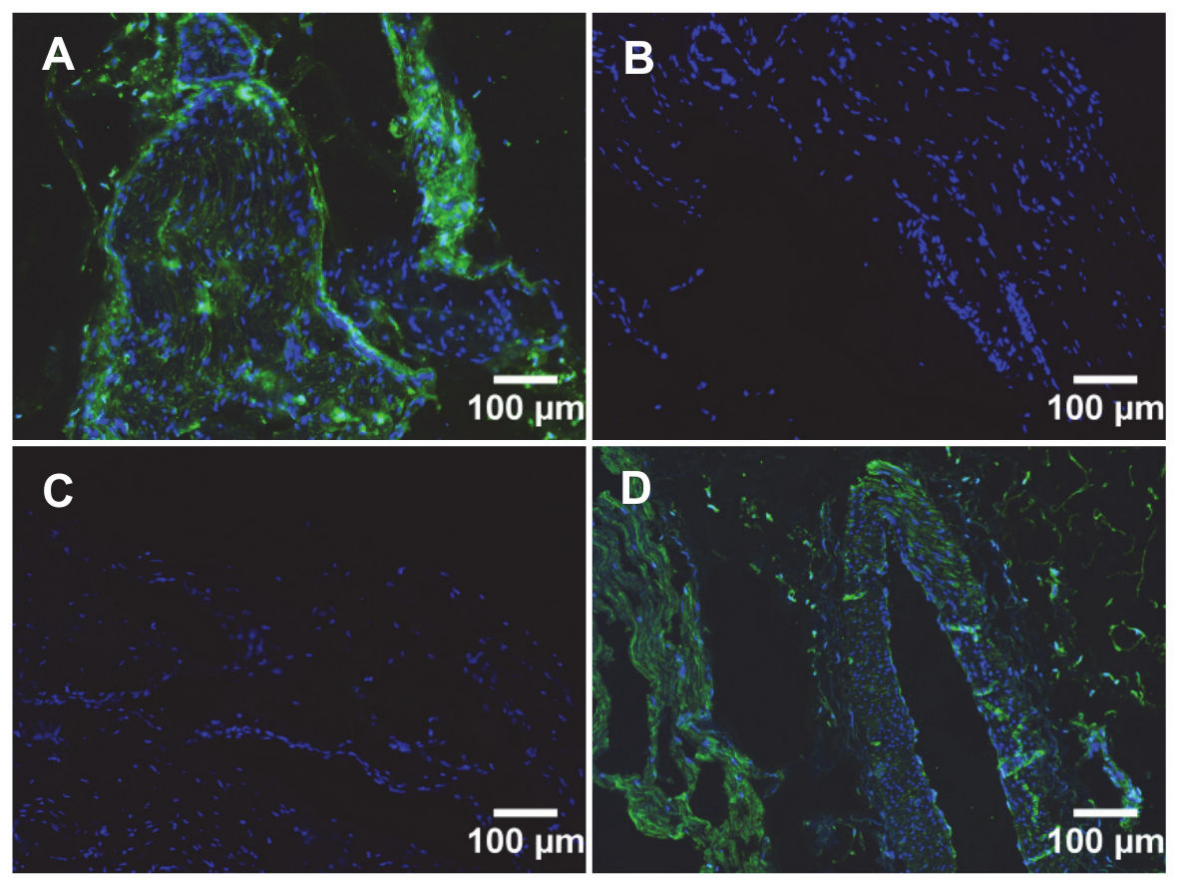
Immunohistochemical analysis of antibody binding to bovine spinal cord tissue. A. B5 scFv detected with mouse anti-hexahistidine antibody and anti-mouse FITC-conjugated antibody. B. As for A but with no anti-hexahistidine antibody. C. As for A but with no B5 scFv. D. Commercial anti-NCAM1 monoclonal antibody. Cell nuclei are stained blue (DAPI) while scFvs attached to the cell surface are stained in green. Scale bar is 100 µm in all images.

The ability of B5 scFv to bind bovine spinal cord and IVD-derived cell NP and AF cells was also confirmed. In immunocytochemical studies, signal intensities were similar for AF and NP cells in repeated analyses (Figure 4B). Similarly, the B5 scFv demonstrated extensive labelling of cells in bovine spinal cord sections (Figure 5). Binding could be successfully reported using the encoded hexahistidine or *myc* (not shown) recombinant tags [25] and the scFv again exhibited a similar labelling pattern to the commercial anti-NCAM1 murine antibody.

**Figure 6:**
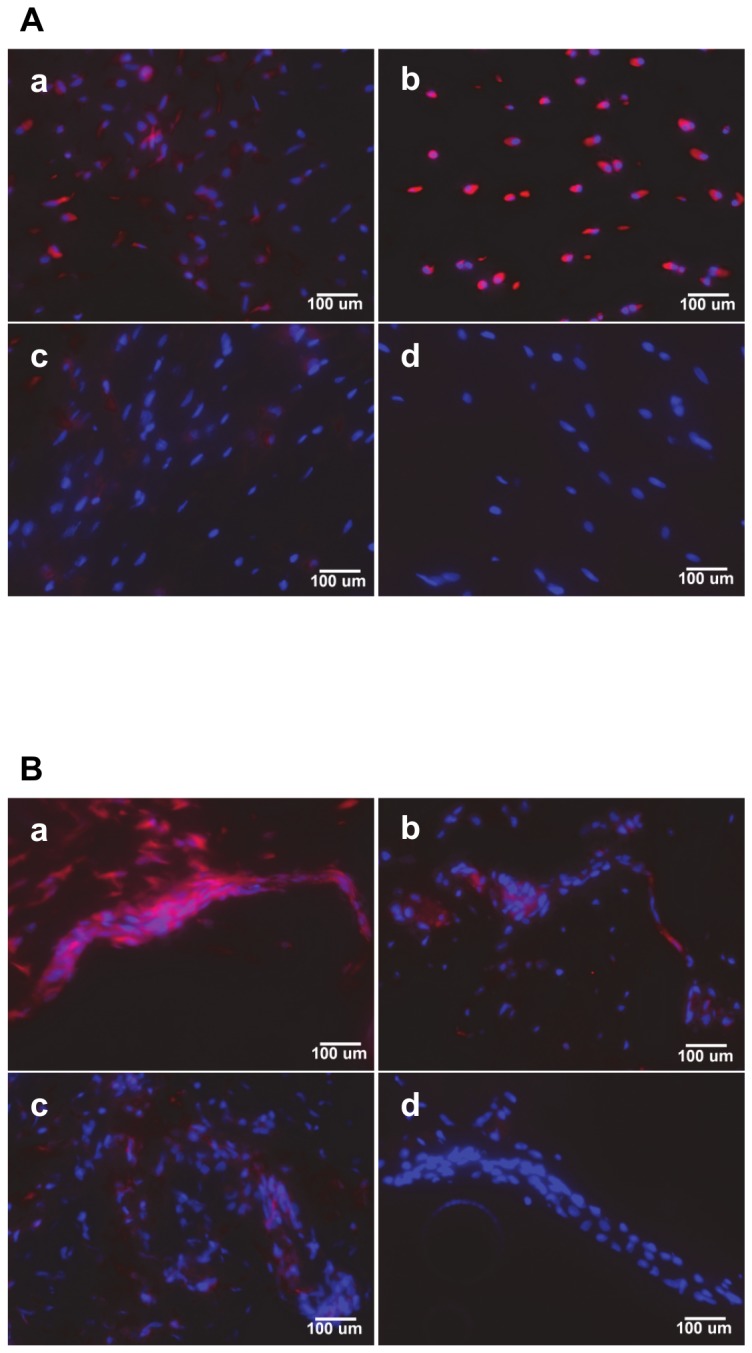
Immunohistochemical analysis of B5 scFv binding to bovine IVD tissues. A: NP tissues. a. Six-month old tissue; b. Two-year old tissue. c,d: as for a,b but with pre-incubation of B5 scFv with 50 µg/ml soluble NCAM-Ig1 domain. B: AF tissues. a. Six-month old tissue; b. Two-year old tissue. c,d: as for a,b but with pre-incubation of B5 scFv with 50 µg/ml soluble NCAM-Ig1 domain. Cell nuclei are stained blue using DAPI while scFvs are stained in pink using a DyLight 568-conjugated reporter antibody. Scale bar is 100 µm in all images.

In the case of IVD tissues, the commercial anti-NCAM1 antibody and B5 scFv demonstrated similar binding patterns (not shown). Meanwhile, the B5 scFv exhibited significant binding to both six-month old and two-year old bovine tissues, and to both NP and AF cells (Figure 6). Higher signal intensities and a higher proportion of labelled cells was observed for NP tissues at two years than six months, while the opposite was the case for AF tissues. In all samples, binding could be inhibited almost completely by the soluble NCAM1-Ig1 domain, indicating the specificity of scFv binding to NCAM1 in the two tissues.

## Discussion

In this study, we describe the isolation of a recombinant antibody fragment that binds neural cell adhesion molecule Ig1 domain for potential application in targeting therapeutics to NCAM1-expressing IVD cells.

Tissue engineering approaches envisaged for the degenerated IVD typically incorporate the addition of healthy cells, reconstitution of the physical and osmotic properties of the disc region *via* a scaffold and the delivery of anabolic, anti-catabolic or anti-inflammatory agents to promote regeneration or delay further degeneration. Progress towards improved therapies has been aided by an enhanced understanding of the pathophysiology of degeneration [38], demonstration of the restorative properties of growth factors such as BMP-2 [39], the development of reservoir systems to deliver cells and simulate the resident tissue [15,40], and the advent of whole organ culture bioreactors to reproduce the mechanical and physiological conditions of the IVD [41,42].

We hypothesise that targeting delivery of therapeutic factors to cells will enhance their efficacies in regenerating or slowing degeneration of naturally resident and newly administered cells in the suboptimal disc environment. This approach takes cognisance of the low cell numbers in the IVD, even before further depletion occurs upon degeneration, which begins as early as their second decade in many individuals. While the IVD region is avascular and contains limited cell types and numbers, cell-specific targeting of therapeutic moieties may clearly also be necessary in more diverse, less contained environments to avoid misplacement of the therapeutic approach *in vivo* as well as to enhance uptake.

The exquisite target specificity of antibodies is commonly exploited to deliver payloads to specific cell types. Traditional monoclonal antibody isolation and production processes, however, are protracted, labour-intensive and expensive. Recombinant antibody-derived fragments, conversely, retain the antigen-binding pocket and, thus, binding ability of whole immunoglobulins but offer advantages of cost, speed and ease of production in expression platforms such as *E. coli*. In addition, they have a greatly reduced size [31,43] (important in, e.g., tissue penetration) and can be readily functionalised with novel properties (e.g. therapeutic or imaging moieties) using readily accessible protein engineering techniques [30,32]. While the increased functionalisation capacity and reduced production costs of scFvs compared with full-length monoclonal antibodies will be advantageous in targeting delivery of biomolecules in the IVD, the avascular microenvironment means that the faster clearance of scFvs is unlikely to be a significant factor, unlike in the case of systemic delivery of therapeutics. Importantly for *in vivo* applications, library approaches such as phage display allow entirely human molecules to be isolated *in vitro*, a breakthrough not yet achieved with mainstream monoclonal antibody technologies.

While NCAM1 is also widely expressed on non-IVD cells, it was selected as an IVD cell marker in this work due to its elevated expression on NP and AF cells compared to articular cartilage cells in the contained IVD micro-environment in canine [23] and human [24] tissues. The cell-distal NCAM1-Ig1 domain was used for antibody isolation to maximise accessibility to cell-targeting scFvs while the previous expression of numerous immunoglobulin superfamily domains in *E. coli* [30,32,44,45] and the distinct modular structure of NCAM1 (Figure 1) increased the likelihood that the Ig1 domain could be expressed in folded, structurally intact form in the bacterial host. Following some expression optimisation, a soluble, recombinant product of the expected molecular weight was purified to near-homogeneity for scFv isolation.

Phage display technology identifies high-affinity antibody fragments against immobilised ligands by mimicking the human B cell response, utilising bacteriophage particles to display antibody fragments [21]. Library panning yielded a phage-scFv with NCAM1-Ig1 binding that could be out-competed by the soluble domain. Fragments with intragenic stop codons were also isolated due to use of an amber-suppressing *E. coli* strain for display of scFvs [25]; these were not investigated further due to their incompatibility with the subsequent expression system.

The ability of the isolated B5 scFv to bind full-length, cell-bound NCAM1 was investigated using rat astrocytes and bovine NP and AF cells. Comparison of NCAM1-Ig1 sequences identified 97% amino acid identity between the human and bovine, and 94% between the human and rat domains (not shown). Immunocytochemical studies confirmed binding of the cell-tethered NCAM1 protein by the recombinant scFv, with similar labelling of rat and bovine cells by the scFv and a commercial anti-NCAM1 antibody. The scFv exhibited higher signal intensities than the monoclonal antibody, albeit using different reporting systems that precluded quantitative comparison of their affinities. Typically, however, antibody fragments from phage libraries can exhibit affinities in the nanomolar range and further affinity improvement is a relatively routine *in vitro* task [46], unlike mutagenesis and isolation of improved monoclonal antibodies [47].

A NP/AF gene expression ratio of around 10 has been reported in beagle tissue [23], beagles proving a good model of human disc degeneration as they lose their notocordal cell population as the young adult ages; conversely, a microarray study noted no difference in NCAM1 expression between human NP and AF tissues [48]. No difference in signal intensities was noted between bovine NP and AF cells upon scFv binding in this work. This labelling pattern indicates that B5 scFv-mediated delivery approaches would target both NP and AF cells in the IVD. As degeneration is hypothesised to begin in the central NP region but with early degenerative processes also evident in the surrounding AF [49,50], this labelling profile is consistent with current treatment practices which target both NP and AF cells affected by the degenerative process to promote production of new ECM or growth factors.

The expression of numerous proteins has been demonstrated to vary in the IVD as natural ageing or accelerated degeneration occur [23,38,48]. The B5 scFv exhibited binding to both six-month and two-year old NP and AF tissues (Figure 6), with higher signals at two years in NP and six months in AF tissues. More detailed investigation of human IVD tissues of different ages and disease grades would be required to determine the ability of the scFv to bind cells throughout the degenerative cycle. The demonstrated ease of isolation and expression of cell-binding scFvs in this study, however, suggests that scFvs against multiple antigens could be co-administered to maximise delivery of therapeutics to IVD cells in distinct degenerative states.

We have demonstrated in this work that a combined phage display and recombinant protein expression approach can be used to isolate scFv antibody fragments that bind surface markers on IVD cells. The described B5 scFv has potential application in targeting of therapeutic moieties to NCAM1-expressing NP and AF cells in aged or diseased IVD. Furthermore, the use of recombinant antibody fragments provides a source of cell-binding molecules for use in tissue engineering applications at significantly lower cost, in reduced times, at higher yields and with greater potential for functionalisation than whole antibodies.

## Supporting Information

Figure S1 **DNA and predicted amino acid sequence of NCAM1 expression construct.** In the DNA sequence, the *Xba*I and *Hind*III restriction sites used for cloning are shown in italics and underlined, the Shine Dalgarno sequence is underlined and the translation start and stop codons are boxed. In the amino acid sequence, the *ompA* leader is highlighted in yellow, the FLAG sequence in teal and the hexahistidine tag in grey.
(DOC)

Table S1 **Oligonucleotides used in the study and predicted sizes of products.**
(DOCX)

## References

[1] Freemont TJ, LeMaitre C, Watkins A, Hoyland JA (2001) Degeneration of intervertebral discs: current understanding of cellular and molecular events, and implications for novel therapies. Expert Rev Mol Med 2001: 1-10 10.1017/S1462399401002885 14987359

[2] Burton AK, Balagué F, Cardon G, Eriksen HR, Henrotin Y et al. (2006) European guidelines for prevention in low back pain: November 2004 chapter 2. Eur Spine J 15: S136-S168. doi:10.1007/s00586-006-1070-3. PubMed: 16550446. 16550446 PMC3454541

[3] Miller JA, Schmatz C, Schultz AB (1988) Lumbar disc degeneration: correlation with age, sex, and spine level in 600 autopsy specimens. Spine (Phila Pa 1976) 13: 173-178. PubMed: 3406837. 3406837

[4] Boos N, Weissbach S, Rohrbach H, Weiler C, Spratt KF et al. (2002) Classification of age-related changes in lumbar intervertebral discs: 2002 Volvo Award in basic science. Spine (Phila Pa 1976) 27: 2631-2644. PubMed: 12461389. 10.1097/00007632-200212010-00002 12461389

[5] Urban JP, Roberts S (2003) Degeneration of the intervertebral disc. Arthritis Res Ther 5: 120-130. doi:10.1186/ar629. PubMed: 12723977. 12723977 PMC165040

[6] Pattappa G, Li Z, Peroglio M, Wismer N, Alini M et al. (2012) Diversity of intervertebral disc cells: phenotype and function. J Anat 221: 480-496. doi:10.1111/j.1469-7580.2012.01521.x. PubMed: 22686699. 22686699 PMC3512276

[7] Roughley PJ (2004) Biology of intervertebral disc aging and degeneration: involvement of the extracellular matrix. Spine (Phila Pa 1976) 29: 2691-2699. PubMed: 15564918. 10.1097/01.brs.0000146101.53784.b1 15564918

[8] Frobin W, Brinckmann P, Kramer M, Hartwig E (2001) Height of lumbar discs measured from radiographs compared with degeneration and height classified from MR images. Eur Radiol 11: 263-269. doi:10.1007/s003300000556. PubMed: 11218025. 11218025

[9] Lyons G, Eisenstein SM, Sweet MB (1981) Biochemical changes in intervertebral disc degeneration. Biochim Biophys Acta 673: 443-453. doi:10.1016/0304-4165(81)90476-1. PubMed: 7225426. 7225426

[10] Kepler CK, Anderson DG, Tannoury C, Ponnappan RK (2011) Intervertebral disc degeneration and emerging biologic treatments. J Am Acad Orthop Surg 19: 543-553. PubMed: 21885700. 21885700 10.5435/00124635-201109000-00005

[11] Prasarn ML, Baria D, Milne E, Latta L, Sukovich W (2012) Adjacent-level biomechanics after single versus multilevel cervical spine fusion. J Neurosurg Spine 16: 172-177. doi:10.3171/2011.10.SPINE11116. PubMed: 22136389. 22136389

[12] Gaetani P, Torre ML, Klinger M, Faustini M, Crovato F et al. (2008) Adipose-derived stem cell therapy for intervertebral disc regeneration: an in vitro reconstructed tissue in alginate capsules. Tissue Eng Part A 14: 1415-1423. doi:10.1089/ten.tea.2007.0330. PubMed: 18593270. 18593270

[13] Strange DGT, Oyen ML (2012) Composite hydrogels for nucleus pulposus tissue engineering. J Mech Behav Biomed Mater 11: 16-26. doi:10.1016/j.jmbbm.2011.10.003. PubMed: 22658151. 22658151

[14] Halloran DO, Grad S, Stoddart M, Dockery P, Alini M et al. (2008) An injectable cross-linked scaffold for nucleus pulposus regeneration. Biomaterials 29: 438-447. doi:10.1016/j.biomaterials.2007.10.009. PubMed: 17959242. 17959242

[15] Collin EC, Grad S, Zeugolis DI, Vinatier CS, Clouet JR et al. (2011) An injectable vehicle for nucleus pulposus cell-based therapy. Biomaterials 32: 2862-2870. doi:10.1016/j.biomaterials.2011.01.018. PubMed: 21276612. 21276612

[16] Richardson SM, Hughes N, Hunt JA, Freemont AJ, Hoyland JA (2008) Human mesenchymal stem cell differentiation to NP-like cells in chitosan–glycerophosphate hydrogels. Biomaterials 29: 85-93. doi:10.1016/j.biomaterials.2007.09.018. PubMed: 17920676. 17920676

[17] Tim Yoon S, Su Kim K, Li J, Soo Park J, Akamaru T et al. (2003) The effect of bone morphogenetic protein-2 on rat intervertebral disc cells in vitro. Spine (Phila Pa 1976) 28: 1773-1780. PubMed: 12923462. 10.1097/01.BRS.0000083204.44190.34 12923462

[18] Hong H, Yang K, Zhang Y, Engle JW, Feng L et al. (2012) In vivo targeting and imaging of tumor vasculature with radiolabeled, antibody-conjugated nanographene. ACS Nano 6: 2361-2370. doi:10.1021/nn204625e. PubMed: 22339280. 22339280 PMC3314116

[19] Asgeirsdóttir SA, Talman EG, de Graaf IA, Kamps JA, Satchell SC et al. (2010) Targeted transfection increases siRNA uptake and gene silencing of primary endothelial cells in vitro--a quantitative study. J Control Release 141: 241-251. doi:10.1016/j.jconrel.2009.09.008. PubMed: 19766679. 19766679

[20] Hu X, O'Connor IB, Wall JG (2012) Antibody immobilization on solid surfaces: methods and applications. In: Syed T Biological Interactions with Surface Charge in Biomaterials. Cambridge, (UK): The Royal Society of Chemistry pp. 90-104.

[21] McCafferty J, Griffiths AD, Winter G, Chiswell DJ (1990) Phage antibodies: filamentous phage displaying antibody variable domains. Nature 348: 552-554. doi:10.1038/348552a0. PubMed: 2247164. 2247164

[22] Rønn LC, Hartz BP, Bock E (1998) The neural cell adhesion molecule (NCAM) in development and plasticity of the nervous system. Exp Gerontol 33: 853-864. doi:10.1016/S0531-5565(98)00040-0. PubMed: 9951628. 9951628

[23] Sakai D, Nakai T, Mochida J, Alini M, Grad S (2009) Differential phenotype of intervertebral disc cells: microarray and immunohistochemical analysis of canine nucleus pulposus and anulus fibrosus. Spine (Phila Pa 1976) 34: 1448-1456. PubMed: 19525835. 10.1097/BRS.0b013e3181a55705 19525835

[24] Rutges J, Creemers LB, Dhert W, Milz S, Sakai D et al. (2010) Variations in gene and protein expression in human nucleus pulposus in comparison with annulus fibrosus and cartilage cells: potential associations with aging and degeneration. Osteoarthritis Cartilage 18: 416-423. doi:10.1016/j.joca.2009.09.009. PubMed: 19833252. 19833252

[25] Pansri P, Jaruseranee N, Rangnoi K, Kristensen P, Yamabhai M (2009) A compact phage display human scFv library for selection of antibodies to a wide variety of antigens. BMC Biotechnol 9: 6. doi:10.1186/1472-6750-9-6. PubMed: 19175944. 19175944 PMC2642811

[26] Thomsen NK, Soroka V, Jensen PH, Berezin V, Kiselyov VV et al. (1996) The three-dimensional structure of the first domain of neural cell adhesion molecule. Nat Struct Biol 3: 581-585. doi:10.1038/nsb0796-581. PubMed: 8673600. 8673600

[27] Guex N, Peitsch MC (1997) SWISS-MODEL and the Swiss-PdbViewer: an environment for comparative protein modeling. Electrophoresis 18: 2714-2723. doi:10.1002/elps.1150181505. PubMed: 9504803. 9504803

[28] Terpe K (2003) Overview of tag protein fusions: from molecular and biochemical fundamentals to commercial systems. Appl Microbiol Biotechnol 60: 523-533. PubMed: 12536251. 12536251 10.1007/s00253-002-1158-6

[29] Studier FW (2005) Protein production by auto-induction in high density shaking cultures. Protein Expr Purif 41: 207-234. doi:10.1016/j.pep.2005.01.016. PubMed: 15915565. 15915565

[30] Hu X, Hortigüela MJ, Robin S, Lin H, Li Y et al. (2013) Covalent and oriented immobilization of scFv antibody fragments via an engineered glycan moiety. Biomacromolecules 14: 153-159. doi:10.1021/bm301518p. PubMed: 23215344. 23215344

[31] Hu X, O'Hara L, White S, Magner E, Kane M et al. (2007) Optimisation of production of a domoic acid-binding scFv antibody fragment in *Escherichia* *coli* using molecular chaperones and functional immobilisation on a mesoporous silicate support. Protein Expr Purif 52: 194-201. doi:10.1016/j.pep.2006.08.009. PubMed: 17005419. 17005419

[32] Hortigüela MW, Wall JG (2013) Improved detection of domoic acid using covalently immobilised antibody fragments. Mar Drugs 11: 881-895. doi:10.3390/md11030881. PubMed: 23493076. 23493076 PMC3705377

[33] Hu X, O'Dwyer R, Wall JG (2005) Cloning, expression and characterisation of a single-chain Fv antibody fragment against domoic acid in Escherichia coli. J Biotechnol 120: 38-45. doi:10.1016/j.jbiotec.2005.05.018. PubMed: 16019098. 16019098

[34] Spada S, Pembroke JT, Wall JG (2002) Isolation of a novel *Thermus* *thermophilus* metal efflux protein that improves *Escherichia* *coli* growth under stress conditions. Extremophiles 6: 301-308. doi:10.1007/s00792-001-0257-0. PubMed: 12215815. 12215815

[35] Newland B, Abu-Rub M, Naughton M, Zheng Y, Pinoncely AV et al. (2013) GDNF gene delivery via a 2-(dimethylamino)ethyl methacrylate based cyclized knot polymer for neuronal cell applications. ACS Chem Neurosci 4: 540-546. doi:10.1021/cn4000023. PubMed: 23391146. 23391146 PMC3630533

[36] O'Dwyer R, Razzaque R, Hu X, Hollingshead SK, Wall JG (2009) Engineering of cysteine residues leads to improved production of a human dipeptidase enzyme in *E.* *coli* . Appl Biochem Biotechnol 159: 178-190. doi:10.1007/s12010-008-8379-9. PubMed: 18931951. 18931951

[37] Altschul SF, Madden TL, Schäffer AA, Zhang J, Zhang Z et al. (1997) Gapped BLAST and PSI-BLAST: a new generation of protein database search programs. Nucleic Acids Res 25: 3389-3402. doi:10.1093/nar/25.17.3389. PubMed: 9254694. 9254694 PMC146917

[38] Sakai D, Nakamura Y, Nakai T, Mishima T, Kato S et al. (2012) Exhaustion of nucleus pulposus progenitor cells with ageing and degeneration of the intervertebral disc. Nat Commun 3: 1264. doi:10.1038/ncomms2226. PubMed: 23232394. 23232394 PMC3535337

[39] Zhang Y, Anderson DG, Phillips FM, Thonar EJ, He TC et al. (2007) Comparative effects of bone morphogenetic proteins and Sox9 overexpression on matrix accumulation by bovine anulus fibrosus cells: implications for anular repair. Spine (Phila Pa 1976) 32: 2515-2520. PubMed: 17978648. 10.1097/BRS.0b013e318158cc09 17978648

[40] Guarino V, Gloria A, Raucci MG, Ambrosio L (2012) Hydrogel-based platforms for the regeneration of osteochondral tissue and intervertebral disc. Polymers 4: 1590-1612. doi:10.3390/polym4031590.

[41] Parolin M, Gawri R, Mwale F, Steffen T, Roughley P et al. (2010) Development of a whole disc organ culture system to study human intervertebral disc. Evid Based Spine Care J 1: 67-68. doi:10.1055/s-0028-1100919. PubMed: 23637672. PMC3623092 23637672

[42] Illien-Jünger S, Gantenbein-Ritter B, Grad S, Lezuo P, Ferguson SJ et al. (2010) The combined effects of limited nutrition and high-frequency loading on intervertebral discs with endplates. Spine 35: 1744-1752. doi:10.1097/BRS.0b013e3181c48019. PubMed: 20395884. 20395884

[43] Hu X, Spada S, White S, Hudson S, Magner E et al. (2006) Adsorption and activity of a domoic acid binding antibody fragment on mesoporous silicates. J Phys Chem B 110: 18703-18709. doi:10.1021/jp062423e. PubMed: 16970501. 16970501

[44] Molloy PE, Graham BM, Cupit PM, Grant SD, Porter AJ et al. (1995) Expression and purification strategies for the production of single-chain antibody and T-cell receptor fragments in *E.* *coli* . Mol Biotechnol 4: 239-245. doi:10.1007/BF02779017. PubMed: 8680930. 8680930

[45] Jendeberg L, Nilsson P, Larsson A, Denker P, Uhlén M et al. (1997) Engineering of Fc(1) and Fc(3) from human immunoglobulin G to analyse subclass specificity for staphylococcal protein A. J Immunol Methods 201: 25-34. doi:10.1016/S0022-1759(96)00215-3. PubMed: 9032407. 9032407

[46] Thie H, Voedisch B, Dubel S, Hust M, Schirrmann T (2009) Affinity maturation by phage display. Methods Mol Biol 525: 309-322, xv 19252854 10.1007/978-1-59745-554-1_16

[47] Chang KH, Kim MS, Hong GW, Seo MS, Shin YN et al. (2012) Affinity maturation of an epidermal growth factor receptor targeting human monoclonal antibody ER414 by CDR mutation. Immune Netw 12: 155-164. doi:10.4110/in.2012.12.4.155. PubMed: 23091439. 23091439 PMC3467414

[48] Power KA, Grad S, Rutges JP, Creemers LB, van Rijen MHP et al. (2011) Identification of cell surface specific markers to target human nucleus pulposus cells: Expression of carbonic anhydrase 12 varies with age and degeneration. Arthritis Rheum 63: 3876-3886. doi:10.1002/art.30607. PubMed: 22127705. 22127705

[49] Gruber HE, Hoelscher GL, Ingram JA, Bethea S, Zinchenko N et al. (2011) Variations in aggrecan localization and gene expression patterns characterize increasing stages of human intervertebral disk degeneration. Exp Mol Pathol 91: 534-539. doi:10.1016/j.yexmp.2011.06.001. PubMed: 21689646. 21689646

[50] Guterl CC, See EY, Blanquer SB, Pandit A, Ferguson SJ et al. (2013) Challenges and strategies in the repair of ruptured annulus fibrosus. Eur Cell Mater 25: 1-21. PubMed: 23283636. 23283636 10.22203/ecm.v025a01 PMC3655691

