# Supplementary material for: Isolation and Characterisation of a Recombinant Antibody Fragment That Binds NCAM1-Expressing Intervertebral Disc Cells
Source: PLoS One. 2013 Dec 13;8(12):e83678. doi: 10.1371/journal.pone.0083678 (PMC3862799; doi:10.1371/journal.pone.0083678)
Supplement: Figure S1 — DNA and predicted amino acid sequence of NCAM1 expression construct. In the DNA sequence, the XbaI and HindIII restriction sites used for cloning are shown in italics and underlined, the Shine Dalgarno sequence is underlined and the translation start and stop codons are boxed. In the amino acid sequence, the ompA leader is highlighted in yellow, the FLAG sequence in teal and the hexahistidine tag in grey. (DOC) [file pone.0083678.s001.doc]

**Supplementary information**

**Figure S1.** **DNA and predicted amino acid sequence of NCAM1 expression construct.**

*XbaI* S/D

1 *TCTAGA*TAACGAGGGCAAAAAATGAAAAAGACAGCTATCGCGATTGCAGTGGCACTGGCT 60
 M K K T A I A I A V A L A

61 GGTTTCGCTACCGTAGCGCAGGCCGACTACAAAGATCTGCAGGTGGATATTGTTCCCAGC 120
 G F A T V A Q A D Y K D L Q V D I V P S

121 CAGGGGGAGATCAGCGTTGGAGAGTCCAAATTCTTCTTATGCCAAGTGGCAGGAGATGCC 180
 Q G E I S V G E S K F F L C Q V A G D A

181 AAAGATAAAGACATCTCCTGGTTCTCCCCCAATGGAGAAAAGCTCACCCCAAACCAGCAG 240
 K D K D I S W F S P N G E K L T P N Q Q

241 CGGATCTCAGTGGTGTGGAATGATGATTCCTCCTCCACCCTCACCATCTATAACGCCAAC 300
 R I S V V W N D D S S S T L T I Y N A N

301 ATCGACGACGCCGGCATTTACAAGTGTGTGGTTACAGGCGAGGATGGCAGTGAGTCAGAG 360
 I D D A G I Y K C V V T G E D G S E S E
 *HindIII*

361 GCCACCGTCAACGGATTGGAAGTACAGGTTCTCCACCACCACCACCACCACTGA*AAGCTT* 420
 A T V N G L E V Q V L H H H H H H
