# Supplementary material for: Isolation and Characterisation of a Recombinant Antibody Fragment That Binds NCAM1-Expressing Intervertebral Disc Cells
Source: PLoS One. 2013 Dec 13;8(12):e83678. doi: 10.1371/journal.pone.0083678 (PMC3862799; doi:10.1371/journal.pone.0083678)
Supplement: Table S1 — Oligonucleotides used in the study and predicted sizes of products. (DOCX) [file pone.0083678.s002.docx]

**Supplementary information**

**Table S1. Oligonucleotides used in the study and predicted sizes of products**

| Oligonucleotide | Sequence | Product length | |
| --- | --- | --- | --- |
| ompA_NCAM1_F | GGTTTCGCTACCGTAGCGCAGGCCCTGCAGGTGGATATTGTTCCCAGCCAGG | **PCR1:**  143 bp |  |
| NCAM1_R | CCAGAGAATTCGTTGACGGTGGCCTCTGACTCACTG |  | Overlap PCR:  402 bp |
| ompA_F | CAGGAAACAGCTATGACCATGATTACG | **PCR2:**  311 bp |  |
| ompA_NCAM1_R | CCTGGCTGGGAACAATATCCACCTGCAGGGCCTGCGCTACGGTAGCGAAACC |  |  |
| scFv sequencing | CAGAGATCAGTTTCTGTTCGG | n/a | |

1 UCUAGAUAACGAGGGCAAAAAAUGAAAAAGACAGCUAUCGCGAUUGCAGUGGCACUGGCU 60
 M K K T A I A I A V A L A

61 GGUUUCGCUACCGUAGCGCAGGCCGACUACAAAGAUCUGCAGGUGGAUAUUGUUCCCAGC 120
 G F A T V A Q A *D Y K D* **L Q V D I V P S**

121 CAGGGGGAGAUCAGCGUUGGAGAGUCCAAAUUCUUCUUAUGCCAAGUGGCAGGAGAUGCC 180
 **Q G E I S V G E S K F F L C Q V A G D A**

181 AAAGAUAAAGACAUCUCCUGGUUCUCCCCCAAUGGAGAAAAGCUCACCCCAAACCAGCAG 240
 **K D K D I S W F S P N G E K L T P N Q Q**

241 CGGAUCUCAGUGGUGUGGAAUGAUGAUUCCUCCUCCACCCUCACCAUCUAUAACGCCAAC 300
 **R I S V V W N D D S S S T L T I Y N A N**

301 AUCGACGACGCCGGCAUUUACAAGUGUGUGGUUACAGGCGAGGAUGGCAGUGAGUCAGAG 360
 **I D D A G I Y K C V V T G E D G S E S E**

361 GCCACCGUCAACGGAUUGGAAGUACAGGUUCUCCACCACCACCACCACCACUGAAAGCUU 420
 **A T V N G L E V Q V L** *H H H H H H* *

**Supplementary information Figure S1.**

Nucleotide and predicted amino acid sequences of the recombinant NCAM1-Ig1 domain expressed in *E. coli*. The human NCAM1-Ig1 amino acid sequence is in bold. The *Xba*I and *Hin*dIII cloning sites are underlined and the AUG start and UGA stop codons are boxed. In the amino acid sequence, the N-terminal *ompA* leader peptide is underlined, the FLAG recognition sequence is underlined and italicised, and the C-terminal hexahistidine tag amino acid sequence is in italics.
